# The global prevalence of oropharyngeal dysphagia in different populations: a systematic review and meta-analysis

**DOI:** 10.1186/s12967-022-03380-0

**Published:** 2022-04-11

**Authors:** Fatemeh Rajati, Nassim Ahmadi, Zahra Al-sadat Naghibzadeh, Mohsen Kazeminia

**Affiliations:** 1 Research Center for Environmental Determinants of Health, Health Institute, Kermanshah University of Medical Sciences, Kermanshah, Iran; 2 Department of Speech Therapy, Rehabilitation Research Center, School of Rehabilitation Sciences, Iran University of Medical Sciences, Tehran, Iran; 3 Medical Biology Research Center, Kermanshah University of Medical Sciences, Kermanshah, Iran; 4 Student Research Committee, Kermanshah University of Medical Sciences, Kermanshah, Iran

**Keywords:** Prevalence, Oropharyngeal dysphagia, Systematic review, Meta-analysis

## Abstract

**Background:**

Oropharyngeal dysphagia (OD) refers to any abnormality in the physiology of swallowing in the upper gastrointestinal tract, which leads to the related clinical complications, such as malnutrition, dehydration, and sever complication, such as aspiration pneumonia, suffocation, and eventually, premature death. The previous studies indicated a various range of prevalence of OD. The present systematic review and meta-analysis aimed to standardize the global prevalence of OD in different populations.

**Methods:**

A systematic literature review was conducted using Embase, Scopus, PubMed, Web of Science (WoS) databases, and Google Scholar motor engine using related MeSH/Emtree and Free Text words, with no time limitation until November 2021. The heterogeneity among studies was quantified using *I*^2^ index and the random effects model was used, due to the high heterogeneity among the results of studies included in the meta-analysis.

**Results:**

The systematic literature search retrieved 2092 studies. After excluding the irrelevant studies, ultimately 27 articles with a sample size of 9841 were included in the meta-analysis. After combining the studies, the overall estimate of the global prevalence rate of OD was 43.8% (95% CI 33.3–54.9%) and the highest prevalence rate was estimated in Africa with 64.2% (95% CI 53.2–73.9%). Given the subgroup analysis based on the study population, the highest prevalence of OD was related to Dementia with 72.4% (95% CI 26.7–95.0%). The results of meta-regression indicated that the prevalence of OD has an increasing trend with the enhancement of year of publication and mean age.

**Conclusion:**

The results of the present systematic review and meta-analysis revealed that the prevalence of OD is high in different populations and its trend has been increasing in recent years. Therefore, the appropriate strategies should be applied to reduce the prevalence of OD by finding its causation and monitoring at all levels, as well as providing feedback to hospitals.

## Introduction

Swallowing is a process requiring the coordination of a complex series of motor, sensory, and psychological activities that are voluntary and involuntary, and most changes in its function occur with aging [1, 2]. Eating and drinking are essential for humans and dysphagia refers to swallowing difficulties [3]. There are different definitions for dysphagia. Given that the International Classification of Functioning, Disability, and Health (ICF) classifies swallowing as “the function of clearing food and drink through the oral cavity, pharynx, and oesophagus (gullet) with an appropriate rate”, dysphagia is defined as: the difficulty in transferring food from the mouth to the stomach [2, 3].

Dysphagia is classified into esophageal dysphagia and oropharyngeal dysphagia [4]. Oropharyngeal dysphagia refers to any abnormality in the physiology of swallowing in the upper gastrointestinal tract [5], including an imbalance in the coordination between the respiratory and nutritional functions [6], and leading to related clinical complications, such as malnutrition, dehydration, and some of the risk factors, such as aspiration pneumonia, asphyxiation, and eventually, premature death [7–9]. Some difficulties, such as loss of muscle mass, changes of the cervical spine, impaired dental status, and reduction of saliva production affect swallowing function. Thus, the risk of OD increases with age and the natural aging processes [10–12].

OD has a variety of causes, including aging, neurological diseases, such as Parkinson’s, dementia, multiple sclerosis, stroke, head and neck cancer, neck surgery, traumatic brain injury, and chronic obstructive pulmonary disease (COPD) [11, 13–15].

OD is associated with symptoms, such as painful swallowing (odynophagia), inability to swallow, sensation of food stuck in the throat or chest or behind the chest, saliva, sniff, reflux, frequent heartburn, acid or food reflux to the throat, unexpected weight loss, coughing or nausea when swallowing, and shrinking food or not eating certain foods, due to swallowing disorders [4–6, 11].

Initial assessments, including video fluoroscopy (VFS) and Fiberoptic Endoscopic Evaluation of Swallowing (FEES) are essential to minimize OD risks [16].

International data reported the prevalence of OD in the general populations between 2.3 and 16.0% [11]. Further, the prevalence of OD is high with predisposing conditions, such as aging and stroke. Its prevalence is reported 26.19% in the elderly [11], 8.1–80% in stroke patients [17], and 21.9–69.5% in patients taking antipsychotic drugs [18].

There are several preliminary studies on the prevalence of OD in different populations in different parts of the world, but these studies examine the prevalence in a small environment and have a smaller sample size. Also, the results of studies showed the different values of the prevalence of this disorder in different populations. None of these studies investigated the effect of potential factors, such as age and prevalence over time, so the present study aimed to standardizing the prevalence of OD in different populations by systematic review and meta-analysis.

## Methods

The present study was conducted according to PRISMA guidelines, including identification, screening, eligibility, and included [19]. The searches, study selection, and data extraction were done independently by two researchers (Z.N. and M.K.) to minimize publication bias and error. Any conflict or disagreement between the two researchers was resolved by the consensus and consultation with a third researcher (F.R.) and the opinion of the third researcher was final.

### Identification of studies

A systematic literature review was conducted using PubMed, Embase, Scopus, and Web of Science (WoS) databases, and Google Scholar motor engine to find out the relevant studies assessing the global prevalence rate of OD in different populations. The searches included the combinations of the following MeSH/Emtree and Free Text words: “Prevalen*”, “Oropharyngeal Dysphagia”, and “Dysphagia Oropharyngeal”. No time limitation was considered for the search to retrieve as comprehensive as possible related studies by November 2021. The references of all included articles and also the studies that cited to the included articles were manually reviewed to maximize the comprehensiveness of the search. Table 1 represents the search strategy of different databases.

**Table 1:** Search strategies

Database	Search strategy	Date	Number
PubMed	((((Prevalence [MeSH Terms]) OR (Prevalen* [Title/Abstract])) OR (Prevalence* [Title/Abstract])) OR (Prevalent [Title/Abstract])) AND (("Oropharyngeal Dysphagia") OR ("Dysphagia, Oropharyngeal"))	14 November 2021	171
Scopus	(TITLE-ABS-KEY (Prevalence*) OR TITLE-ABS-KEY (Prevalence*) OR TITLE-ABS-KEY (*Prevalent*)) AND (ALL ("*Dysphagia*, *Oropharyngeal*") OR ALL ("*Oropharyngeal Dysphagia*"))	16 November 2021	839
WoS	TS=(Prevalence* OR Prevalence OR Prevalent) AND ALL=(“Oropharyngeal Dysphagia” OR “Dysphagia, Oropharyngeal”)	16 November 2021	462
Embase	#1: 'prevalence*':ab,ti OR 'Prevalence*':ab,ti OR 'prevalent':ab,ti OR 'prevalence'/exp/mj #2: 'oropharyngeal dysphagia' #3: #1 AND #2	17 November 2021	370
Google scholar	(Prevalence* OR Prevalence OR Prevalent) AND (“Oropharyngeal Dysphagia” OR “Dysphagia, Oropharyngeal”)	18 November 2021	250

### Inclusion criteria

The inclusion criteria were original scientific-research articles, observational studies, access to the full text of the article, and studies reported the prevalence rate of OD.

### Exclusion criteria

The exclusion criteria included the irrelevant studies, cross-sectional studies, case reports, case series, papers presented at conferences, letter to the editor, qualitative studies, dissertations, systematic review and meta-analysis, animal studies, and lack of access to the full text of the articles.

### Selection process of studies

All articles derived from various databases were imported into EndNote X8 software. After eliminating the duplicates, the title and abstract of the studies were thoroughly screened to excluded the irrelevant studies. The full text of remaining articles was carefully assessed for eligibility and irrelevant studies were removed. Finally, the quality assessment of the studies met inclusion criteria was done. Researchers extracted the articles without knowing the name of authors, institutes, and journals.

### Qualitative evaluation of the studies

The quality assessment of studies was done using the Joanna Briggs Institute (JBI) checklist for prevalence studies [20], which consists of 9 different items, including sample frame, participants, sample size, study subjects and the setting described in detail, data analysis, valid methods for identifying conditions, measuring the situation, statistical analysis, and response rate adequate. The sources of bias were identified using the criteria that the reviewers qualified with answers, including yes, no, unclear, or not applicable. The sum of “yes” scores was calculated to evaluate each study. Therefore, the total score range based on the number of “yes” is between 0 and 9.

### Data extraction

A pre-prepared electronic checklist was employed to extract the data. The items of this checklist included first author, year of publication, country, sample size, age, study design, diagnostic tools, prevalence rate, and quality assessment score.

### Statistical analysis

The prevalence rate of OD was reviewed in this study and the frequency rate of OD, i.e., the frequency of patients suffered from OD was divided by the total number of subjects in each study to combine the results of different studies. The heterogeneity of studies was checked using *I*^2^ index and due to the high heterogeneity between the results of the studies included in the meta-analysis (*I*^2^ ˃ 75%), the random effects model was applied, which calculates the parameter changes between studies. Thus, the results of random effects model in heterogeneous conditions are more generalizable than those of fixed effect model. Funnel plot and Begg and Mazumdar rank correlation were used to assess the publication bias. In addition, meta-regression was used to examine the relationship between the global prevalence rate of OD and the year of publication, sample size, and mean age. The subgroup analysis was performed according to different continents (Asia, Europe, USA, Africa, and Australia), study population, and type of diagnostic tool. The comprehensive meta-analysis software (version 2) was applied for meta-analysis and P-value less than 0.05 was regarded as statistically significant.

## Results

### The summary of how studies included in the meta-analysis

**Fig. 1:**
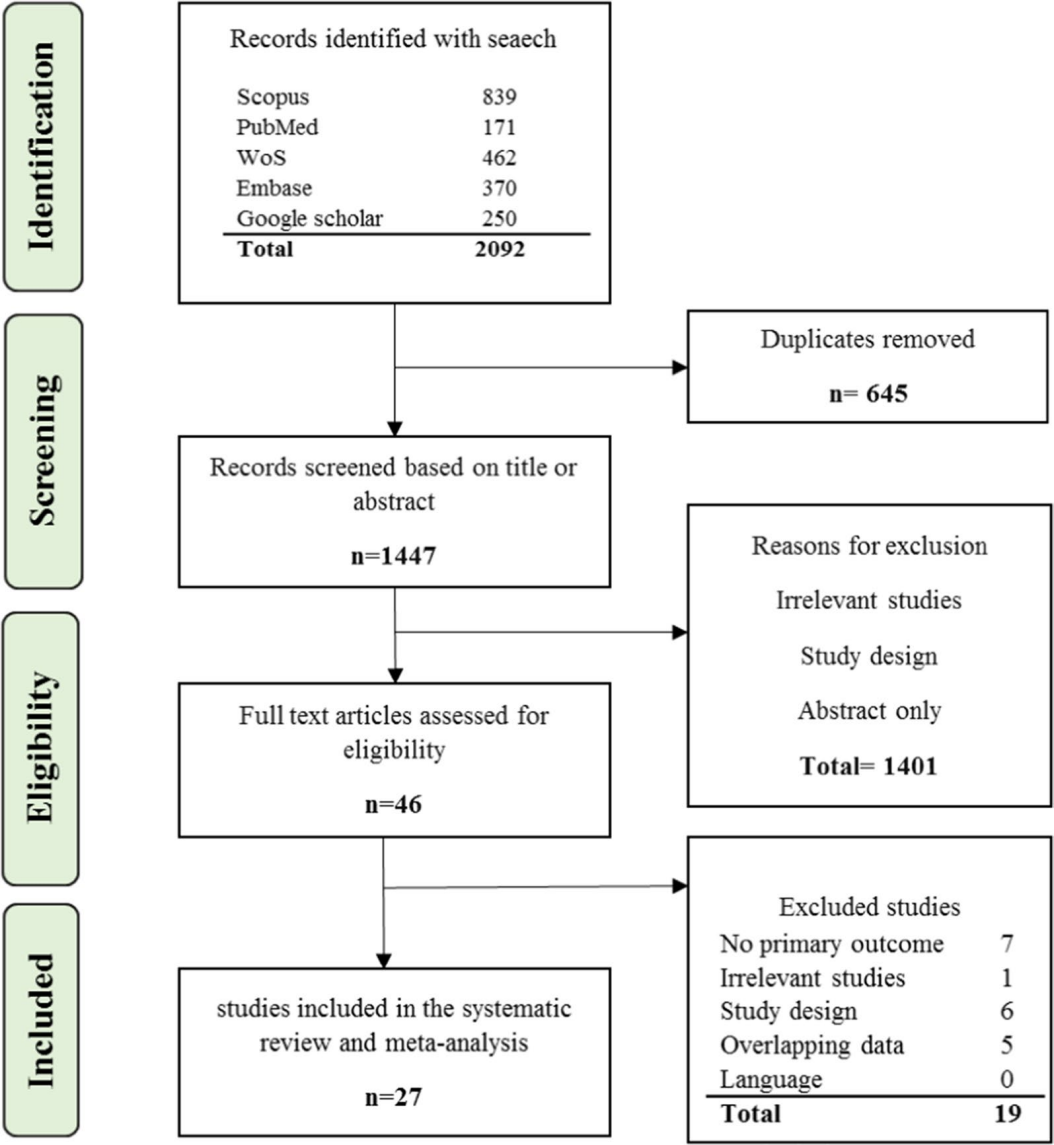
PRISMA 2020 flow diagram for article selection

In the initial search, 2092 studies were identified. After eliminating 645 duplicates and studies with overlapping data, 1401 irrelevant studies were removed by screening the title and abstract. Then, full text of the remaining 46 studies were inspected carefully and 19 articles were excluded due to not meeting eligibility criteria. Finally, 27 articles met inclusion criteria were included in the meta-analysis. Figure 1 displays the PRISMA 2020 flow diagram.

### General characteristics of the studies

The total sample size was 9841. The oldest study was performed in 1991 and the most recent study in 2021. The highest number of studies was conducted in Spain with 7 articles. The maximum and minimum sample size was related to the study of David et al. [21] with 2973 subjects and the study of Almeida et al. [22] with 25, respectively. The diagnostic tool for OD in most studies was physical examination (12 articles) or volume–viscosity swallow test (10 articles). The highest quality assessment score based on the JBI checklist was related to the study of Wolf et al. [23] with a score of 9. Table 2 represents the characteristics of studies included in the systematic review and meta-analysis.

**Table 2:** The characteristics of the studies included in the systematic review and meta-analysis

First author, year, (References)	Country (continent)	Sample size (n)			Age (year)	Type of study	Diagnostic tool	Prevalence (%)			Population	Quality score
		Total	Male	Female				Total	Male	Female		
Wolf, 2020, [23]	Germany (Europe)	200	69	131	84 ± 6.5	Cross-sectional retrospective	Physical examination	29.0	–	–	Elderly	9
Ruth, 1991, [24]	Sweden (Europe)	337	–	–	20–79	Cross-sectional	Physical examination	10.0	–	–	General population	4
Cabre, 2010, [25]	Spain (Europe)	134	80	54	84.51 ± 6.8	Prospective cohort	Physical examination	55.0	–	–	Elderly patients with pneumonia	5
Melgaard, 2017, [26]	Denmark (Europe)	154	84	70	80.90 ± 10.58	Cross-sectional observational	Volume–viscosity swallow test	34.42	47.61	17.14	Elderly patients with community-acquired pneumonia	7
Michel, 2018, [27]	France (Europe)	117	40	77	84.5 ± 5.1	Prospective study	Volume–viscosity swallow test	86.6	95.0	81.81	Older patients with dementia	7
Elvira, 2020, [28]	Spain (Europe)	255	98	157	83.5 ± 8	Prospective longitudinal quasi-experimental	Volume–viscosity swallow test	85.9	85.35	61.2	Older patients with dementia	8
Holland, 2011, [29]	UK (Europe)	637	149	488	89 (69–98)	Longitudinal study	Swallow questionnaire	11.4	–	–	Elderly	5
Garcı´a-Peris, 2007, [30]	Spain (Europe)	87	–	–	58.2 ± 13.5	Cross-sectional retrospective	Physical examination	50.6	–	–	Head and neck cancer	8
Rofes, 2018, [31]	Spain (Europe)	395	211	184	73.2 ± 13.3	Cohort	Volume–viscosity swallow test	45.06	38.86	52.2	Stroke	7
Mateos-Nozal, 2020, [32]	Spain (Europe)	329	104	225	93.5 (81–106)	Observational prospective	Volume–viscosity swallow test	82.4	87.5	70.8	Elderly	8
Falsetti, 2009, [33]	Italy (Europe)	151	77	74	79.4 ± 6.2	Cross-sectional retrospective	Volume–viscosity swallow test	26.5	40.25	12.16	Stroke	4
Lendinez-Mesa, 2017, [34]	Spain (Europe)	124	88	36	56.45 ± 12.35	Cross-sectional	Physical examination	79.3	–	–	Stroke	6
Serra-Prat, 2012, [35]	Spain (Europe)	254	136	118	77.4 ± 5.0	Population-based prospective study	Volume–viscosity swallow test	18.6	9.5	28.8	Elderly	6
Hamdy, 2014, [36]	UK (Europe)	180	93	87	74.2 ± 11.5	Cross-sectional	Volume–viscosity swallow test	41.7	–	–	Stroke	4
Stefano, 2020, [37]	Italy (Europe)	708	367	341	75.9 ± 8.6	Cross-sectional retrospective	Physical examination	32.7	–	–	Older patients with dementia	7
Melgaard, 2018, [38]	Denmark (Europe)	313	138	175	83.1 ± 7.8	Cross-sectional observational	Volume–viscosity swallow test	50.0	52.17	48.0	Acute Geriatric Patients	8
Lindh, 2017, [39]	Sweden (Europe)	51	–	–	48.3 ± 6.3	Observational prospective	Physical examination	49.0	–	–	COPD	7
David, 2008, [21]	Australia (Australia)	2973	–	–	49.4 (15–95)	Population-based prospective study	Physical examination	7.3	–	–	General population	6
Yang, 2013, [40]	Korea (Asia)	415	195	220	77.3 ± 8.7	Cohort	Swallow questionnaire	33.7	39.5	28.6	Elderly	7
Biglary, 2019, [41]	Iran (Asia)	500	–	–	48.1 ± 7.5	Cross-sectional study was a descriptive-analytic study	Physical examination	17.39	–	–	Neurological diseases and head and neck surgery	5
Costa, 2019, [42]	South African (African)	81	–	–	11.7 ± 15.6 day	Prospective cross-sectional observational	Clinical feeding assessments	64.2	–	–	Neonates	7
Chiocca, 2005, [43]	Argentina (America)	839	373	466	39.9 ± 15.4	Cross-Sectional Observational	Physical examination	29.6	–	–	General population	7
Jacinto-Scudeiro, 2019, [44]	Brasil (America)	36	6	30	34.7 ± 16.8	Cross-sectional	Swallow questionnaire	33.0	–	–	Paraplegia	5
Delevatti, 2020, [45]	Brasil (America)	229	49	180	77.90 ± 8.21	Cross-sectional	Volume–viscosity swallow test	58.0	49.0	60.5	Older adults with orthopedic fractures	6
Almeida, 2015, [22]	Brasil (America)	25	–	–	62 (44–80)	Descriptive retrospective	Physical examination	96.0	–	–	Stroke	4
Samantha, 2015, [46]	Colorado (America)	206	9	187	32 (23–47)	Large retrospective review	Swallow questionnaire	20.38	44.44	20.32	Patients with severe anorexia nervosa	5
Benfer, 2018, [47]	USA (America)	111	82	29	34.1 ± 11.9 month	Longitudinal population-based cohort	Physical examination	79.7	–	–	Children with cerebral palsy	5

### Meta-analysis of the global prevalence of OD

**Fig. 2:**
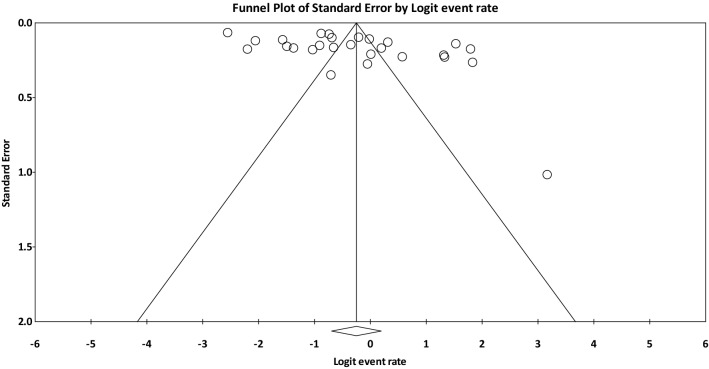
The Funnel plot of the results of the overall estimation of the global prevalence of OD

**Fig. 3:**
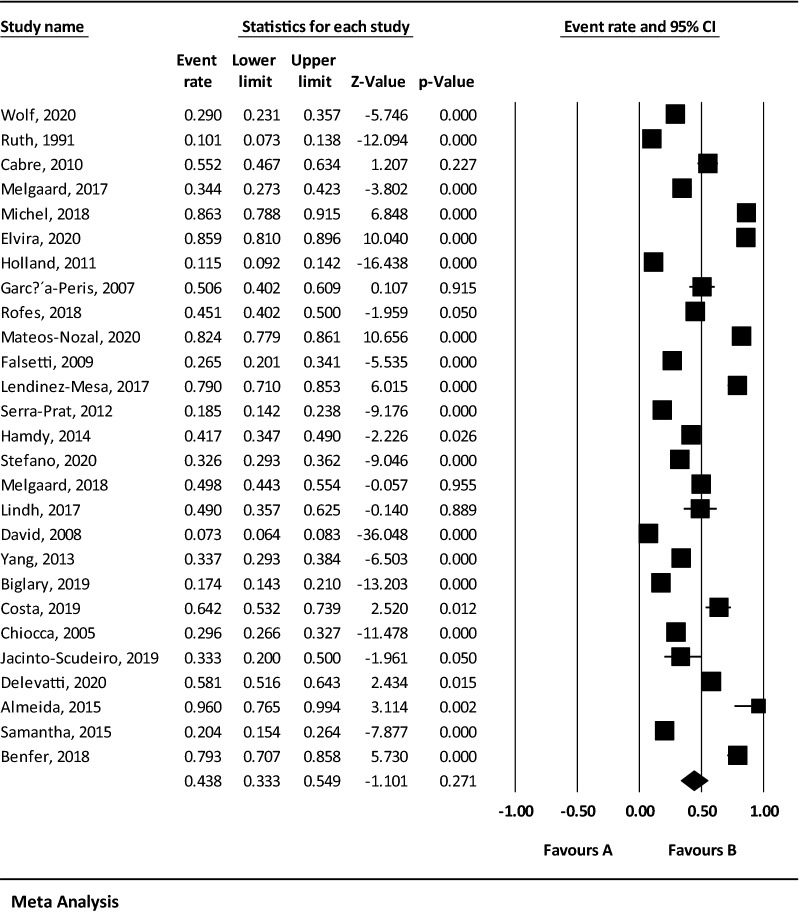
The forest plot of the overall estimation of the global prevalence of OD based on the random effects model

**Fig. 4:**
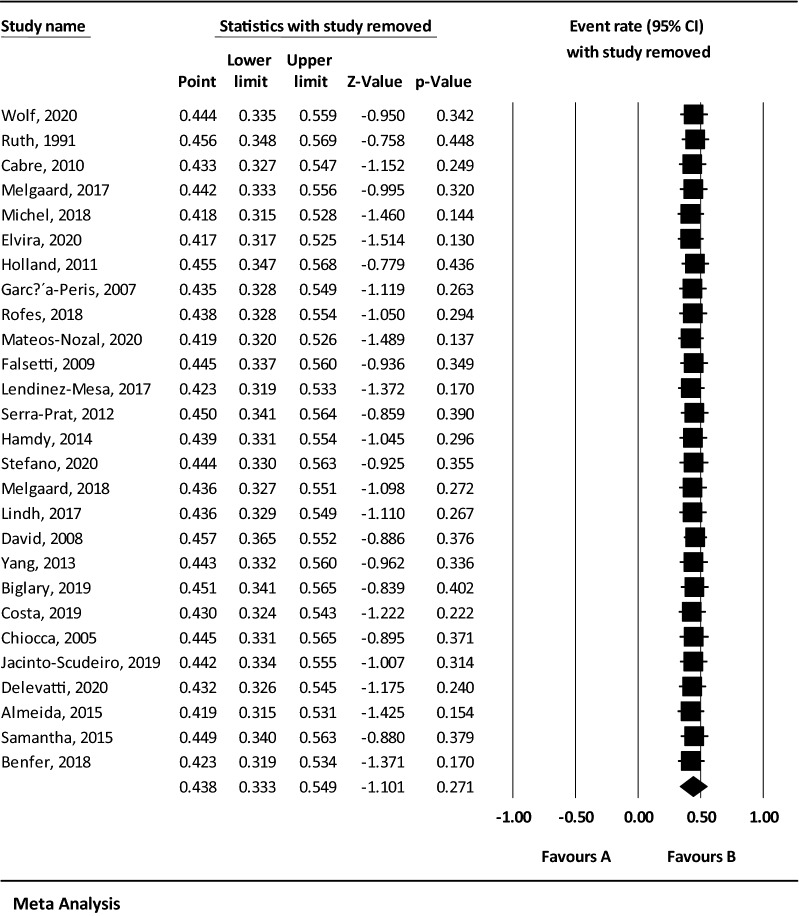
The sensitivity analysis chart of the global prevalence of OD based on the random effects model

Considering that the result of *I*^2^ test for the global prevalence of OD indicated a significant heterogeneity among included studies (*I*^2^ = 98.60), the data were analyzed using a random effects model (Table 3). Based on the results of Begg and Mazumdar rank correlation, there was no publication bias at the level of 0.05 in the studies (*P*-valve = 0.103) (Fig. 2). As a result of combining the results of studies, the overall estimate of the global prevalence of OD was 43.8% (95% CI 33.3–54.9%) based on the random effects model. As shown in the Fig. 3, the black square represents the prevalence rate, the length of the line segment displays the 95% CI in each study, and the rhombus symbol illustrates the global prevalence rate of OD for all studies. The results of sensitivity analysis demonstrated that the pooled estimation did not change significantly by removing any of the studies (Fig. 4).

**Table 3:** Reporting the results of fixed and random effects model on meta-analysis

Model	Number studies	Point estimate	Lower limit	Upper limit	Z-value	P-value	Q-value	Df (Q)	P-value	I-squared	Tau squared	Standard error	Variance	Tau
Fixed	27	0.316	0.305	0.327	− 29.611	0.000	1859.987	26	0.000	98.602	1.339	0.501	0.251	1.157
Random	27	0.438	0.333	0.549	− 1.101	0.271

### The meta-regression of the global prevalence of OD

**Fig. 5:**
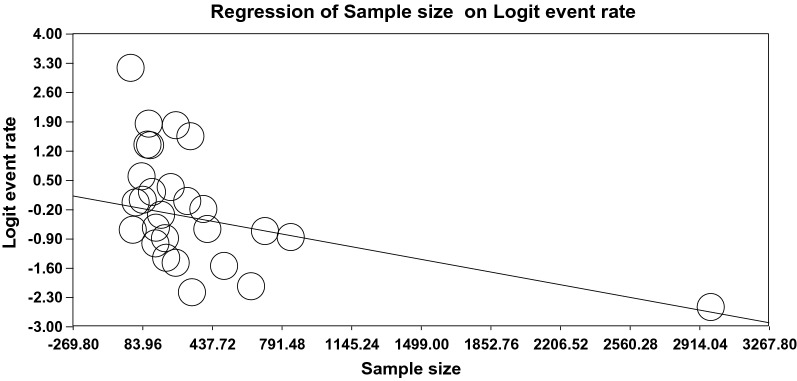
The meta-regression of the relationship between sample size and the global prevalence of OD

**Fig. 6:**
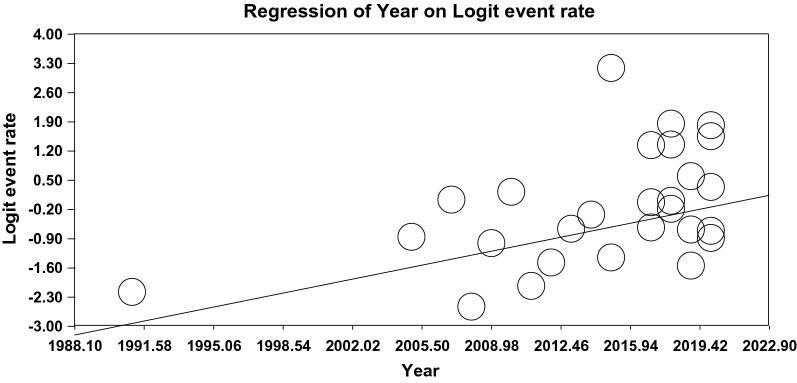
The meta-regression of the relationship between the year of the publication and the global prevalence of OD

**Fig. 7:**
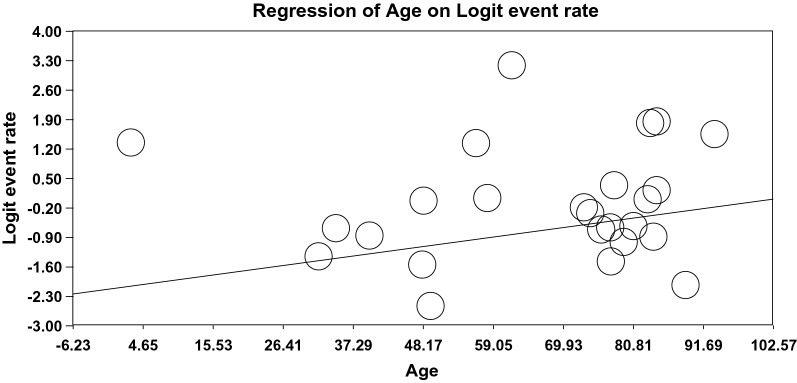
The meta-regression of the relationship between the mean age and the global prevalence of OD

The relationship between the sample size (Fig. 5), year of the publication (Fig. 6), and mean age (Fig. 7) and the global prevalence of OD was assessed using meta-regression. The results indicated a significant difference between the global prevalence of OD and these potential factors (*P* < 0.001). Since the global prevalence of OD decreased by increasing sample size and this prevalence enhanced by increasing the year of the publication and mean age (Figs. 5, 6, 7).

### Subgroup analysis

Given the high heterogeneity among the studies (*I*^2^ = 98.60), subgroup analysis was employed based on the continent, diagnostic tool, study population, and gender (Table 4). The results of the subgroup analysis illustrated that the highest prevalence rate of OD was related to the African continent with 64.2% (95% CI 53.2–73.9%), diagnostic tool of volume–viscosity swallow test with 54.4% (95% CI 39.2–68.8%), patients suffering from dementia with 72.4% (95% CI 26.7–95.0%), and men with 54.7% (95% CI 40.1–68.6%) (Table 4).

**Table 4:** The subgroup analysis of estimating the prevalence rate of OD based on the continents, diagnostic tool, and study population

Subgroups	Number of articles	Sample size	*I*^2^	Begg and Mazumdar	Prevalence % (95% CI)
Continents					
Asia	2	227	96.82	–	24.7 (95% CI 12.1–43.7)
Europe	17	1777	98.12	0.433	45.7 (95% CI 33.3–58.5)
America	6	1446	96.90	0.452	51.3 (95% CI 31.7–70.6)
African	1	81	0.000	–	64.2 (95% CI 53.2–73.9)
Australia	1	2973	0.000	–	7.3 (95% CI 6.4–8.3)
Diagnostic tools					
Physical examination	12	6089	98.69	0.243	40.9 (95% CI 26.3–57.3)
Volume–viscosity swallow test	10	1273	97.76	1.000	54.4 (95% CI 39.2–68.8)
Swallow questionnaire	3	255	97.21	1.000	20.4 (95% CI 9.6–38.4)
Population					
Children	2	192	81.10	–	72.3 (95% CI 55.5–84.6)
Adults	8	4816	98.69	0.386	32.6 (95% CI 17.7–52.0)
Elderly	11	1400	98.56	0.119	48.1 (95% CI 31.9–64.7)
General population	3	4149	99.25	1.000	13.4 (95% CI 4.4–34.5)
Pneumonia	2	288	91.92	–	44.6 (95% CI 25.8–65.0)
Dementia	3	1080	99.10	1.000	72.4 (95% CI 26.7–95.0)
Head and neck cancer	2	587	97.60	–	31.5 (95% CI 8.9–68.4)
Stroke	5	875	94.96	0.806	55.4 (95% CI 37.2–72.2)
Gender					
Male	11	1141	94.36	0.161	54.7 (95% CI 40.1–68.6)
Female	11	1667	97.00	0.876	46.5 (95% CI 31.3–62.5)

## Discussion

The present systematic review and meta-analysis study aimed to estimate the global prevalence of OD in different populations. After combining the data from 27 articles, the global prevalence of OD was estimated to be 43.8%. The highest prevalence rate of OD (96%) was reported in the study of Almeida et al. [22] and the lowest rate (7.3%) in the study of Watson and Lally [21]. The highest quality assessment score based on JBI checklist criteria was related to the study of Wolf et al. [23], which reported the prevalence rate of OD as 29%.

Kertscher et al. reported the prevalence of OD in the Netherlands between 2.3 and 16% [11]. Further, the prevalence of OD was estimated between 8.1 and 80% in stroke patients, 11–81% in the Parkinson’s disease, 27–30% in the traumatic brain injury patients, and 91.7% in the community-acquired pneumonia in the systematic review study of Takizawa et al. [17]. The findings of the present study are not consistent with the results of the afore-mentioned systematic review or meta-analysis studies, which can be attributed to the high number of articles included in the present study (27 articles versus 6 articles in the study of Kertscher et al.). Further, the study of Kertscher et al. examined the studies conducted in the Netherlands while the present study included people with different races and geographies around the world, and the present study was conducted as a systematic review and meta-analysis, while the study of Takizawa et al. was done only systematically and they did not perform statistical analysis.

Considering the results of the meta-regression, the prevalence of OD showed an increasing trend by increasing the mean age. Additionally, the results of subgroup analysis demonstrated that the prevalence of OD is high in the elderly population. Kertscher et al. reported that the prevalence of OD in the population over 75 years old is more than other age groups [11], which is consistent with the results of the present study. Many physiological changes occur in body tissue with aging, such as muscle wasting, reduced endurance capacity, and muscle weakness [48, 49], hormonal changes and decreased ratio of anabolic to catabolic hormones [50], increased rates of neurological diseases [51–53], cardiovascular diseases [54], atrophy of the pharyngeal and laryngeal muscles [55], and many other chronic diseases. Considering these conditions in the treatment process and the improvement of the clinical outcomes of the elderly population can be helpful.

The results of the present study also showed that the prevalence of OD in the pediatric population is high. Although the number of studies investigated in the pediatric population was small (2 articles), the reasons for this could be abnormalities or dental problems, large tongue and tonsils, problems with prenatal development of cranial bones and structures of the mouth and throat (known as Craniofacial abnormalities), prenatal abnormalities of the gastrointestinal tract, such as esophageal atresia (esophageal obstruction) or tracheovasophageal fistula after prolonged exposure to a ventilator (which may occur in premature infants or very sick children), vocal cord paralysis, tracheostomy surgery, esophageal stimulation or ulceration due to gastric acid in gastroesophageal reflux disease. Esophageal obstruction by other body structures, such as enlarged heart, thyroid gland, blood vessels or lymph nodes, growth retardation, and prematurity of the baby [42, 47].

The results of subgroup analysis revealed that the prevalence of OD in patients with dementia is higher than that in other study population. Dementia is a chronic disease with a set of symptoms, such as memory impairment, language impairment, psychological changes, and behavioral disorders [56]. When dementia reaches its advanced stages, brain changes lead to the dysfunction of organs and physical activities, such as swallowing disorder, dysphagia, loss of balance, and incontinence [57, 58]. Dementia is a global challenge that directly affects 47.5 million people worldwide and 7.7 million people each year [59]. Taking into account these conditions and complications can assist the treatment process and improve the quality of life of patients with dementia.

Based on the results of the present study, the prevalence of OD is high in stroke, which is in line with the results of the systematic review study of Takizawa et al. [17]. Meng et al. reported that the prevalence of swallowing disorders was 36.3% (95% CI 33.3–39.3%) in patients with stroke [60]. Stroke is a sudden neurological disorder, resulting in impaired blood flow to the area affected by the stroke. In other words, when blood flow to a part of the brain is disrupted and stopped, that part can no longer function normally [61, 62]. The post-stroke complications, depending on the location of the stroke and the extent of brain tissue affected [61], can be vision problems, memory problems, dysphagia (paralysis of the muscles of the pharynx, tongue or mouth), lack of coordination between the eyes and hands, difficulty in decision making, lack of body temperature control, difficulty breathing, urinary and fecal incontinence, nervous system problems, tromboemboli, heart failure, depression, etc. [63–66]. Therefore, it is recommended that health care providers and policy makers pay more attention to the stroke prevention and post-stroke complications, especially OD.

Due to the variation of the population structure in different countries of the world, it was necessary to carefully study the prevalence of OD in different continents in order for planners to pay more attention to the process and its consequences. Therefore, according to the subgroup analyses based on the different continents, the highest prevalence of OD was related to the African continent with 64.2% and the lowest was related to Australia with 7.3%.

The high prevalence of OD in different populations, especially in the elderly and patients with dementia and stroke in the present systematic review and meta-analysis study reveals the need for the investigation and follow-up of OD disorder. Due to the complications of OD and its significant impact on various aspects of life, health care providers and policy makers should pay special attention to the prevalence of OD. Accordingly, we should be aware of OD, find and implement suitable solutions, and follow the results of the measures at the individual, group, and organizational levels to reduce its prevalence.

One of the strengths of this study was estimating the global prevalence of OD for the first time in different populations with a sample size above 9000 people and estimating prevalence of OD in continents and various diagnostic tools. In addition, high heterogeneity among studies (more than 95%) led us to perform subgroup analysis, which reduced a small amount of heterogeneity. However, there is still a lot of heterogeneity in all subgroups, which may be due to the sample size, demographic characteristics, and method.

The present study comes with some limitations, including the lack of uniform reporting of articles, non-random selection of some samples, non-uniform study design, and the lack of access to the full text of articles presented at conferences. Furthermore, the number of studies performed on some populations was limited, therefore, it is suggested to conduct further studies on some patients, such as patients with pneumonia, head and neck cancer, paraplegia, children, etc.

## Conclusion

The results of the present study indicated that the prevalence of OD is high in different populations and its trend has been increasing in recent years. Therefore, the appropriate strategies should be employed to decrease the prevalence of OD by finding its causation and monitoring at all levels, as well as providing feedback to hospitals.

## Data Availability

Datasets are available through the corresponding author upon reasonable request.

## Abbreviations

OD: Oropharyngeal dysphagia
WoS: Web of Science
MeSH: Medical subject headings
PRISMA: Preferred Reporting Items for Systematic Reviews and Meta-Analysis
